# Calcium pyrophosphate deposition disease: historical overview and potential gaps

**DOI:** 10.3389/fmed.2024.1380135

**Published:** 2024-04-04

**Authors:** Carlos Pineda, Hugo Sandoval, Iván Pérez-Neri, Carina Soto-Fajardo, Fabián Carranza-Enríquez

**Affiliations:** ^1^Instituto Nacional de Rehabilitación Luis Guillermo Ibarra Ibarra, Mexico City, Mexico; ^2^Evidence Synthesis Unit, Instituto Nacional de Rehabilitación Luis Guillermo Ibarra Ibarra, Mexico City, Mexico; ^3^Department of Rheumatology, Instituto Nacional de Rehabilitación Luis Guillermo Ibarra Ibarra, Mexico City, Mexico

**Keywords:** chondrocalcinosis, Pseudogout, calcium pyrophosphate, crystal arthropathies, CPPD

## Abstract

CPPD disease can affect patients’ quality of life through its various clinical presentations. This mini-review discusses the evolution of CPPD from its discovery to current knowledge of its pathogenesis, genetic associations, diagnostics, and treatment options. Despite extensive research, the exact mechanisms of CPPD are not well understood, and there is a notable lack of knowledge about psychosocial impacts and patient experiences. This study aims to present a CPPD Disease Timeline identifying gaps in current knowledge and potential directions for future research. These findings contribute to a broader understanding of CPPD disease and emphasize the importance of continued research and innovation in this field.

## Introduction

Calcium Pyrophosphate Deposition (CPPD) Disease occurs when calcium pyrophosphate dihydrate (CPP) crystals are deposited in the articular cartilage and periarticular tissues. This condition is of concern in rheumatology because of its potential impact on a patient’s quality of life (1). CPPD disease is a clinically heterogeneous condition that can present in several forms, including acute calcium pyrophosphate (CPP) crystal arthritis, osteoarthritis (OA) with CPPD disease, chronic CPP crystal inflammatory arthritis, and crowned dens syndrome. The most commonly cartilage calcification associated with CPPD and detected by imaging or histology is referred to as chondrocalcinosis (CC) (2). It affects various anatomical sites, including but not limited to the knee, wrist, hip, spine, and temporomandibular joint (2–4).

Radiographic identification and terminology began to be developed in the 1920s. Familial forms were introduced in the late 1950s. The relationship with calcium pyrophosphate crystals was finally established in the 1960s, leading to the term CPPD, which encompasses all cases of calcium pyrophosphate crystals (CPP) in joints and periarticular tissues.

Consequently, advances in imaging techniques, genetic discoveries, and ongoing insights into novel treatment and management strategies have marked the late 20th and early 21st centuries. This mini-review highlights both the historical background and recent milestones in CPPD disease, including innovative imaging modalities and genetic associations with this entity, such as the ANKH gene, focusing on potential gaps and perspectives.

**Figure 1:**
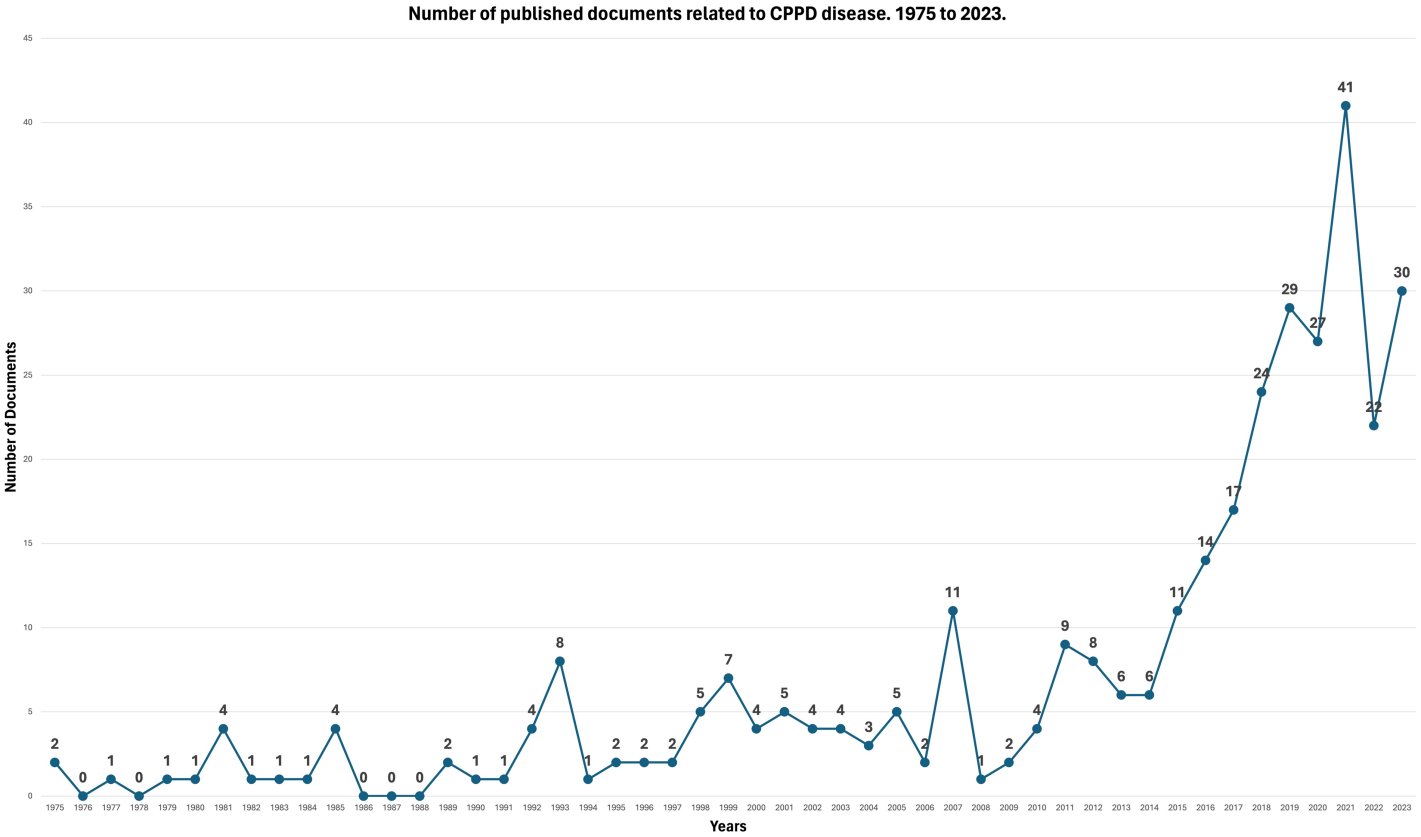
Number of published documents related to CPPD disease 1975–2023 (Source: Scopus).

Figure 1 shows the number of published documents related to CPPD disease recorded per year in the Scopus database from 1975 to 2023. There has been a gradual increase in this number over the years, particularly in the last decade, albeit with some fluctuations; resulting in well-defined peaks and troughs, indicating higher and lower periods of research activity or publication frequency. Since 2014, there has been a noticeable increase in this trend, resulting in a steeper slope. The highest peak occurred in 2021, indicating the maximum number of documents registered. This year was followed by a decrease in 2022 and a partial recovery in 2023.

Although research on CPPD disease began several decades ago and was mainly held by Italian and Anglo-Saxon authors, in recent years an extensive collaborative group has been integrated with participation of many centers throughout the globe.

## CPPD disease timeline

### 19th century

**1842**. Roderick McLeod identified a white powdery substance on the surfaces of articular cartilage during autopsies, probably representing the first observation of chondrocalcinosis (5, 6).

**1873.** Robert Adams published a detailed description of chondrocalcinosis in a patient with chronic rheumatic disease. At autopsy, he observed calcification of the fibrocartilage of the knee meniscus. His description provides valuable insights into the pathogenesis and clinical manifestations of this condition (7).

### Early 20th century

In its early stages, CPPD disease was often misdiagnosed as rheumatoid arthritis or osteoarthritis because of overlapping clinical features. However, radiographic techniques made it possible to visualize calcium deposits within the cartilage.

**1922.** Jacques Calvé and Marcel Galland documented the observation of calcification in intervertebral discs that were visible on radiographs (8).

**1927.** Felix Mandl describes articular cartilage calcifications seen radiographically (9).

**1960–62.** Dusan Zitnan and Stefan Sitaj coined the term “chondrocalcinosis articularis,” which was followed in 1962, by a detailed description of radiographic calcification of the articular cartilage of members of several Slovak families with chronic oligoarthritis. This was the first report of a familial cluster of chondrocalcinosis (10, 11).

### Late 20th century

**1962.** Daniel McCarty and colleagues found non-urate crystals in the affected joints of two patients (12). In one case, the typical clinical features of acute gouty arthritis were present, but the serum urate level was normal. Following this observation, the researchers then examined under the microscope many samples of synovial fluid removed from so-called arthritic joints under the microscope. They were able to identify non-urate crystals in six additional cases (13).

Additionally, McCarty’s group identified and characterized the presence of calcium pyrophosphate crystals in the synovial fluid of patients with acute synovitis using polarized light microscopy. They established the crystal structure of CPPD and definitively distinguished this clinical entity from other joint diseases based on the nature of the crystals. The authors named the disease as “pseudo-gout” due to its close resemblance to an acute gouty attack (14).

**1969.** The term “pyrophosphate arthropathy” was introduced to accurately describe the structural joint changes that occur in CPPD disease (15, 16).

**1975.** The term “calcium pyrophosphate deposition disease” was formally introduced for the first time by various groups of investigators (17, 18). Further descriptions of deposition in diverse anatomical structures were made in subsequent years (3).

**1979.** Original diagnostic criteria for CPPD disease were proposed by Ryan and McCarty (19).

**1989.** Magnetic resonance imaging emerged as an innovative method for assessing calcium deposition in periarticular tissues, beginning with the description of two separate case reports with involvement of cervical vertebrae (20, 21).

**1995.** First description of sonographic findings of CPPD in the knee joints of 28 patients with documented CC (22). Ultrasonography showed linear hyperechoic features in the condylar cartilage, parallel to the bone surface, which were interpreted as calcifications because of their agreement with conventional radiography.

**1997.** A clinical trial demonstrated improvement in pain with the use of hydroxychloroquine in patients with chronic CPPD (23).

### Early 21st century

**2001–2002.** Advances in molecular genetics led to the discovery of ANKH, the first gene associated with the CPPD disease phenotype. The ANKH protein has been implicated in pyrophosphate metabolism, specifically in the transport of pyrophosphate from the intracellular to the extracellular compartment (19–21).

**2000s.** Multiple studies have been carried out revealing the prevalence and incidence of chondrocalcinosis in different age groups and specific population groups. However, epidemiological studies of this entity are yet to be carried out in the general population (24–31).

**2006.** Using an “*in vivo*” rat model, CPP crystals are shown to cleave the caspase-1-activating NALP3 inflammasome, which in turn stimulates production of active IL-1B and IL-18 (32).

**2008.** Clinical studies increasingly began to focus on the potential use of anakinra, an IL-1 receptor antagonist. In the treatment of CPPD disease (33).

**2009.** Use of highly sensitive methods, such as Raman spectroscopy, for the identification of CPP crystals (34–37).

### 2010s–present

**2011.** European League Against Rheumatism recommendations for CPPD terminology, diagnosis, and management, mainly focusing on symptom management through pharmacological interventions including NSAIDs and corticosteroids (2, 38).

**2011.** Ultrasound (US) was identified as a highly accurate tool for detecting CPP crystal deposition in musculoskeletal structures, with both high sensitivity and specificity (39).

**2014.** A randomized controlled trial failed to prove a substantial benefit of methotrexate for treating CPDD disease (40–42); however, observational studies showing more positive results leave an open question on the role of this drug for the treatment of CPPD disease.

**2018.** A gain-of-function mutation in TNFRSF11B encoding osteoprotegerin was found to cause familial CPPD disease, suggesting a bone pathology contribution (43).

**2018.** Preliminary studies on targeted therapies aimed at modulating pyrophosphate metabolism as a treatment modality (44).

**2018**. The OMERACT group published the new sonographic definitions of elementary lesions in CPPD disease (45). Likewise, the good intra- and inter-observer reliability of US by using these definitions was proven (46).

**2019.** Development of novel imaging techniques, such as dual-energy CT scans, continue to favor a better understanding of the disease, improve diagnosis and distinguish CPPD among other forms of microcrystalline deposits (47).

**2020.** A systematic literature review supports the use of anakinra as a therapeutic option for patients with CPPD disease, especially in cases of acute refractory CPPD or when standard treatments are contraindicated (48).

**2020.** Publication of results of the first clinical trials employing biologic drugs for the treatment of CPPD (49, 50), including tocilizumab in symptomatic patients.

**2021.** Development of Machine Learning approaches and Electronic Health Record Data algorithms for the identification of CPPD (51).

**2022.** Classifying Pseudogout using Machine Learning Approaches with Electronic Health Record Data. An approach to the identification of acute subtype of CPPD disease patients using electronic health record data with a positive predictive value of 81% was developed (52).

**2022.** The first Consensus-based definitions of imaging features characteristic of CPPD on CR, CT, DECT, US and MRI served as a reference for future clinical research studies and diagnostic criteria (53).

**2023.** Publication of the ACR/EULAR CPPD disease classification criteria, which showed very good diagnostic performance and provided guidance for future research (54).

**2023.** A clinical trial demonstrates equivalence between colchicine and prednisone for the treatment of acute calcium pyrophosphate crystal arthritis (55).

**Figure 2:**
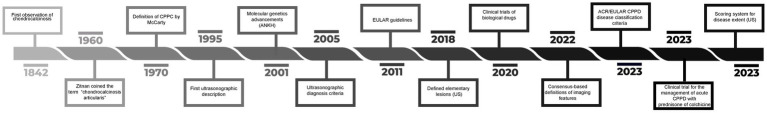
CPPD timeline.

**2023.** Development and validation by the OMERACT group of a scoring system to determine the extent of CPPD by US (56). Figure 2 shows a synthetized timeline highlighting the most relevant events listed in the past section.

## Identified gaps

### Epidemiology and demographics

Although there is a considerable amount of data on the prevalence and incidence of CPPD and chondrocalcinosis, particularly in older populations, there appears to be a gap in detailed epidemiological studies focusing on specific demographics such as younger populations or specific ethnic groups. Understanding the prevalence and characteristics of these diseases in different populations could provide valuable insights into the genetic and environmental risk factors.

### Pathogenesis and molecular mechanisms

The exact mechanisms underlying CPPD and chondrocalcinosis are not fully understood. Research into the cellular and molecular pathways involved in crystal formation and deposition may lead to a better understanding of the potential therapeutic targets. Studies into the role of ANKH mutations and their influence on CPPD are particularly promising.

### Diagnostic tools and biomarkers

There is ongoing research to improve the diagnostic methods for CPPD and chondrocalcinosis; however, there is still a need for more accurate, non-invasive, and early detection tools. Identification of specific biomarkers associated with these conditions could improve diagnostic accuracy and help monitor disease progression.

### Treatment and management

Although there are available treatments to manage the symptoms and complications of CPPD and chondrocalcinosis, of specific therapies targeting the underlying causes of these diseases are lacking. Research into new pharmacological agents or therapeutic approaches that may alter course of the disease or prevent crystal deposition is a potential key intervention that remains unresolved. Similarly, although some small clinical trials have shown treatment benefits with drugs such as hydroxychloroquine, anakinra, or tocilizumab for chronic management or colchicine or prednisone for acute management, an update of new treatment guidelines is needed.

### Genetic studies and familial patterns

While familial patterns and genetic links have been identified, particularly with the ANKH gene, more comprehensive genetic studies could provide deeper insights into the hereditary aspects of these diseases. This could lead to a better risk assessment and targeted interventions for individuals with a family history of CPPD or Chondrocalcinosis.

### Longitudinal studies

Long-term studies of patients with CPPD and chondrocalcinosis could provide valuable data on the natural history, progression, and long-term outcomes of these diseases. This could improve our understanding of the disease course and improve the management strategies.

### Comorbidity and multimorbidity research

Exploring the relationship between CPPD, chondrocalcinosis, and other comorbid conditions (such as osteoarthritis, cardiovascular diseases, and additional associated metabolic disorders) could provide important insights into the broader health impact of these conditions.

### Patient-centered research

There is a need for more research focusing on patient experiences, quality of life, and psychosocial aspects of living with CPPD and chondrocalcinosis. This includes understanding the impact on daily activities, mental health, and the effectiveness of different support systems.

## Discussion

In conclusion, major advances have been made in the field of CPPD disease since its first description almost two centuries ago, beginning with its first categorization as a separate crystal arthropathy from gout more than six decades ago. Specific areas to be highlighted because of their direct current impact on clinical practice are the development of alternative imaging tools (with a greater diagnostic accuracy than conventional radiography), the first validated classification criteria with international endorsement and an ongoing exhaustive research activity on the underlying pathogenetic mechanisms that trigger and maintain calcium deposition among tissues.

Other fields, like the description of several therapeutic strategies in chronic and acute presentations of CPPD disease, or the discovery of new genetic mutations directly linked to it, though still far from having considerable impact over its natural history, hold promising for further improving how we understand disease pathogenesis in the oncoming years; future research must be directed toward improving long-term clinical outcomes.

## Author contributions

CP: Conceptualization, Data curation, Investigation, Methodology, Writing – original draft, Writing – review & editing. HS: Conceptualization, Data curation, Investigation, Methodology, Writing – original draft, Writing – review & editing. IP-N: Conceptualization, Data curation, Investigation, Writing – original draft, Writing – review & editing. CS-F: Conceptualization, Data curation, Investigation, Writing – original draft, Writing – review & editing. FC-E: Conceptualization, Data curation, Investigation, Methodology, Writing – original draft, Writing – review & editing.
